# Supplementary material for: Exploring discrepancies between in vivo and simulated correction in 3D-planned distal radius osteotomies: the influence of biological factors
Source: PLoS One. 2026 May 26;21(5):e0350018. doi: 10.1371/journal.pone.0350018 (PMC13210242; doi:10.1371/journal.pone.0350018)
Supplement: Attachment — Submitted filename: Response to reviewers.docx [file pone.0350018.s004.docx]

Dear Editor and Reviewers,

On behalf of all authors, we sincerely thank you for your valuable comments and constructive suggestions. We believe that the manuscript has been substantially improved following careful consideration and revision in response to all comments.

In this response letter, we cite the comments in full and refer to the comments using the following notation: EC1 denotes Editor Comment 1, and R1C1 denotes Reviewer 1, Comment 1, and so on. All corresponding revisions in the manuscript have been clearly indicated using this convention.

**Editor’s comments:**

When submitting your revision, we need you to address these additional requirements.

**EC1**.Please ensure that your manuscript meets PLOS ONE's style requirements, including those for file naming. The PLOS ONE style templates can be found at

https://journals.plos.org/plosone/s/file?id=wjVg/PLOSOne_formatting_sample_main_body.pdf and

<https://journals.plos.org/plosone/s/file?id=ba62/PLOSOne_formatting_sample_title_authors_affiliations.pdf>

**Response to EC1:** We apologise for not following the style requirements. We ensure that the revised manuscript is formatted and edited in accordance with these requirements.

**EC2.** Please note that funding information should not appear in any section or other areas of your manuscript. We will only publish funding information present in the Funding Statement section of the online submission form. Please remove any funding-related text from the manuscript.

**EC2 Response:** The funding information was deleted from the manuscript.

**EC3.** Your ethics statement should only appear in the Methods section of your manuscript. If your ethics statement is written in any section besides the Methods, please delete it from any other section.

**EC3 Response:** We reviewed the manuscript and ensured that the ethics statement is included only in the Methods section.

**EC4.** If the reviewer comments include a recommendation to cite specific previously published works, please review and evaluate these publications to determine whether they are relevant and should be cited. There is no requirement to cite these works unless the editor has indicated otherwise.

**EC4 Response:** Thank you for this clarification.

Comments to the Author

**Reviewer 1 Comments**

**R1C1:** The manuscript investigates how bone mineral density and planned maximum distraction influence residual correction errors in 3D-planned distal radius osteotomies performed with 3D virtual surgical planning (3D VSP) and patient-specific surgical guides (PSSGs). The topic is clinically relevant and contributes to imporving accuracy in corrective osteotomy. However, some issue should be considered and addressed.

**R1C1 Response:** Thank you for your positive assessment of our study and the constructive and detailed comments.

**R1C2 Comment.** The objective is not clearly stated and there is no hypothesis.

**R1C2 Response:** Thank you for pointing this out. We agree that the objective was not clear. While reading the reviewers’ comments, we realised that the aims were also too broad. As for the hypothesis, our study was exploratory; we did not include any hypothesis.

In response to this comment, we clarified the objective and narrowed it to analysing discrepancies between in vivo and simulated 3D-planned distal radius osteotomy and determining whether soft tissue tension and bone density are associated with these discrepancies. We edited the Title and final paragraph of the Background section as follows.

The Title was edited to:

“*Discrepancies between in vivo and simulated correction in 3D-planned distal radius osteotomies: the influence of biological factors.”*

The final paragraph of the Background was edited to:

“*The aim of this exploratory study was to assess discrepancies between in vivo and simulated correction in 3D-planned distal radius osteotomy and to examine whether these discrepancies were associated with soft tissue- and bone density -related factors. Specifically, we explored the associations of age, bone mineral density, and planned distraction with discrepancies in volar tilt (VT), radial inclination (RI), and radial length (RL) between the simulated and in vivo surgery. We also examined the differences and agreement in residual errors between in vivo and simulated corrections.”*

**R1C3 Comment**. The term “clinical parameters” is not optimal, and it is not clear from the outset that a comparison will be made. It could be replaced by “influence of soft tissue tension and bone density.”

**R1C3 Response:** Thank you for this suggestion. We replaced “clinical parameters” with “influence of soft tissue tension and bone density” throughout the manuscript.

**R1C4 Comment**. The hypothesis could refer specifically to the comparison between actual surgery and surgery performed on 3D models.

**R1C4 Response:** As we explained in R1C2 Response, our study was exploratory, therefore we abstained from testing a hypothesis.

**R1C5 Comment:** In terms of methodology, this would also mean better identification and description of the two groups: “actual surgery” and “surgery on 3D models” in a dedicated chapter.
**R1C5 Response:** Thank you for this suggestion. We agree with the reviewer. We have revised the Methods section to clearly define and distinguish the two groups:

•	In vivo group (IV): correction achieved during the operative procedure.

•	Simulation on 3D-printed models group (SIM): correction achieved during simulated surgery performed on patient-specific 3D-printed models based on the preoperative 3D virtual surgical plan (VSP).

These groups are now described in a dedicated subsection of the Methods, including workflow, measurement procedures, and data acquisition. See R1C7 for details, where we further review the Methods section.

**R1C6 Comment:** The rationale for comparing simulated and actual surgeries should be more explicitly linked to the clinical question.

**R1C6 Response:** We agree that a more explicit link to the clinical question improves methodological transparency. We have revised the Introduction to more clearly link the comparison between simulated and actual surgeries to the clinical question. The manuscript now explicitly states:

“*However, simulated osteotomies on 3D-printed models do not reproduce important biological conditions present during actual surgery, including bone quality and soft-tissue tension. As a result, the correction achieved in simulation may differ from the correction achieved in vivo. The magnitude of this difference, and whether it is associated with factors related to bone density or soft-tissue tension, has not been established. This limits the external validity of simulation-based studies using 3D-printed models.”*

Following the updates to the manuscript, we also included a new analysis that was originally missing, but which aligns with the clarified objective:

*“Assessment of the discrepancy between IV and SIM*

*The discrepancy between IV and SIM corrections was calculated as the relative rigid transformation between the two models. Specifically, the transformation mapping SIM to IV was computed as:*

$T_{SIM\to IV}=T_{IV\to3D VSP}^{-1}\cdot T_{SIM\to3D VSP}$*,*

*where* $T_{IV\to3D VSP}$ *and* $T_{SIM\to3D VSP}$ *represent the rigid transformations of the IV and SIM models relative to the 3D VSP, respectively. The inverse of the IV transformation was used to express the SIM model in the IV coordinate frame. From the resulting relative transformation matrix, differences in VT (°), RI (°), and RL (mm) between IV and SIM were calculated.”*

Methods
**R1C7.** The methods should be divided in different chapters with subtitle.

**R1C7 response:** Thank you for this helpful suggestion. We have reorganized the Methods section into clearly defined subsections to improve transparency and reproducibility. The revised structure now includes:

*Study design, and participant cohort*

*Image and virtual model acquisition*

*Biological and clinical variables*

*Assessment of in vivo (IV) correction accuracy*

*Assessment of simulated (SIM) correction accuracy*

*Assessment of discrepancy between IV and SIM*

*Statistical analysis*

To ensure structural consistency, the Results section was reorganized to mirror the analytical framework of the Materials and methods section.

**R1C8**. Line 20: It would be clearer and more illustrative to include a figure showing how the measurements were obtained

**R1C8 Response:** We agree that this is important. We included a figure illustrating how the measurements were done.

*“Fig 1. Bone mineral density measurement according to Wagner et al. [11] . A circular region of interest was placed within the cancellous bone of the ulnar head. Measurements were obtained on three consecutive coronal CT slices A-C at the level of the distal radioulnar joint and averaged for analysis.”*

**R1C9.** De la même façon, il manque des images du modèle 3D utilisé et avec la reduction.

Translation: Similarly, images of the 3D model used and with the reduction are missing.

**R1C9 Response:** Following the reviewer’s suggestion, we have included a figure illustrating representative 3D models used in this study.

*“Fig 2. Representative example of the virtual models used for analysis in one patient. (A) Preoperative 3D virtual surgical plan. (B) Postoperative model derived from CT imaging after surgery (in vivo result). (C) Model obtained from the simulated corrective procedure performed on the 3D-printed bone (simulation result).”*

**R1C10.** Line 2 (p5): why “consecutive”? So specify the design of the study anad the period of the inclusion

**R1C10 Response:** Thank you for this comment. We used the term “consecutive” to indicate that all eligible patients undergoing corrective osteotomy for extra-articular distal radius malunion during the specified study period were included without selection.

The participants were derived from a prospective cohort previously reported by Stor Swinkels et al. (reference 9). We have now clarified this in the Methods section and explicitly stated the study design and inclusion period as follows:

“*This retrospective observational study represents a secondary analysis of a prospectively recruited consecutive cohort of patients who underwent corrective osteotomy for extra-articular distal radius malunion at Sahlgrenska University Hospital* *between 13 July 2021 and 13 July 2023. The cohort was originally enrolled as part of a previously published prospective study (Stor Swinkels et al. [9]).*”

**R1C11.** Line 16 (p5): Wagner method, why did the author choose this method ?

**R1C11 Response:** Thank you for this important question. Conventional dual-energy X-ray absorptiometry (DEXA) measurements were not available for this cohort, and therefore an alternative method for estimating bone mineral density was required.

We selected the approach described by Wagner et al. because it provides a validated method for estimating bone mineral density using Hounsfield unit (HU) measurements from CT scans. Importantly, Wagner et al. demonstrated that HU measurements obtained from the distal ulna correlate with bone mineral density and can serve as a reliable proxy in clinical studies. This anatomical region was available in all preoperative CT scans and is adjacent to the surgical site, making it relevant for our investigation.

We have clarified this rationale in the Methods section:

“*As dual-energy X-ray absorptiometry (DEXA) measurements were not available for this cohort, bone mineral density was estimated from preoperative CT scans using Hounsfield unit (HU) measurements. We applied the method described by Wagner et al. (13), who demonstrated that HU values obtained from the distal ulna correlate with bone mineral density and may serve as a surrogate measure in clinical studies. The distal ulna was selected because it was consistently included in all CT scans and is anatomically adjacent to the surgical site. The mean HU value from three consecutive coronal slices was used for analysis (Fig 1).*”

**R1C12.** Line 1 (p6): specify the units (VT, RI, RL…)

**R1C12 Response:** We added units for each parameter throughout the manuscript.

**R1C13.** Line 1 (p7): difference was expressed in SIM-TRU or TRU-SIM ?

**R1C13 Response:** Thank you for highlighting this important point. The discrepancy between IV and SIM was computed as a rigid transformation that maps SIM onto IV. This transformation was derived from the respective rigid transformations of IV and SIM relative to the 3D VSP reference frame. We have clarified this explicitly in the Methods subsection “*Assessment of the discrepancy between IV and SIM*” to ensure transparency and consistency.

In the Bland-Altman agreement analysis, the difference between the methods is calculated as SIM-IV. We clarified it in the caption showing the Bland-Altman plot:

*“Bland-Altman plot of the difference in residual errors between the simulated (SIM) surgery on 3D-printed models and in vivo (IV) postoperative models (SIM – IV)”.*

Results

**R1C14**. Line 1-3 (p9): Add a short explanation of the clinical relevance thresholds

**R1C14 Response:** Thank you for this suggestion. We expanded the explanation of the clinically relevant thresholds that we use in our study for volar tilt and radial length. In the Methods, we wrote:

*“**Bias and limits of agreement in VT and RL analysis were compared to values below which the errors are considered clinically irrelevant: 5° for VT and 2 mm for RL [13–16].“*

We edited the Results as follows:

*“The upper limit of agreement exceeds the clinically relevant threshold of 5°, which has been reported as the minimum clinically relevant change in volar tilt [13–16].”*

**R1C15.** Line 8-14 (p9): Provide more interpretation, especially since some limits of agreement exceed clinically acceptable bounds.

**R1C15 Response:** According to this suggestion, we expanded the description of the outliers that contribute to the large limits of agreement:

*“The residual VT error in IV for that participant was -8.0°, while in SIM – 3.0°. The IV error could be attributed to bone density and soft tissue tension, as the patient had low BMD (11 HU and osteopenia noted in the patient’s journal) and the planned maximum distraction was large: 13.7 mm.“*, and:

*“Thus, the lower limit of agreement for RL slightly exceeded the clinically relevant difference of 2 mm [13–16]. Again, this is due to differences in one patient who had an IV residual RL error of 2 mm, while the SIM residual error was -0.5mm. This patient had high BMD (158 HU), and the source of the residual error is not obvious.”*

Discussion
**R1C16.** Line 25 (p11): “3D-printing errors affect the guide and the bone model” please specify

**R1C16 Response:** Thank you for this comment. We have clarified the statement in the Discussion to specify the potential sources of 3D-printing–related inaccuracies.

Specifically, 3D-printing may introduce (1) dimensional deviations due to printer resolution limits, (2) surface irregularities affecting the fit of patient-specific surgical guides on the printed bone model, and (3) drilling in the printed models during simulated surgery may cause localized surface deformation or enlargement of drill holes, which could influence subsequent guide seating or fixation accuracy.

These clarifications have been added to the Discussion to improve transparency regarding potential sources of discrepancy between simulated and in vivo corrections:

“*Although simulated procedures eliminate biological variability, they are not entirely free of technical sources of error. Dimensional deviations may arise from printer resolution limits or material shrinkage, while surface irregularities of the printed models may affect the fit of the PSSGs. In addition, drilling and fixation of guide wires during simulated procedures may cause localized material deformation, which could affect the accuracy of subsequent guide fixation.*”

**R1C17.** Conclusion: too long, should be only related to the findings

**R1C17 Response:** Thank you for pointing this out. We have edited the conclusions of this study as follows:

“*This exploratory study evaluated discrepancies between simulated and in vivo distal radius osteotomies performed using patient-specific surgical guides and examined whether these discrepancies were associated with bone mineral density and soft-tissue–related factors. Simulated osteotomies on 3D-printed bone models produced residual correction errors broadly comparable to those observed in vivo. However, discrepancies in radial length were associated with planned distraction and bone mineral density, suggesting that biological factors absent from simulation models may influence surgical outcomes. These findings support the use of simulation models for comparative studies while emphasizing the need to consider biological factors when interpreting results from simulation-based evaluations.”*

**R1C18.** Remove : “Understanding how bone quality influences the surgical outcomes will enable the development of predictive models for correction loss, allowing for improved surgical planning and optimized 3D techniques. Further studies are needed to understand errors in simulated surgeries to make it a robust research method in 3D VSP and PSSG”

**R1C18 Response.** We followed the reviewer’s suggestion and removed the text.

**Reviewer 2 Comments**

The authors have provided a manuscript of their study analyzing the influence of bone mineral density and planned distraction on distal radius osteotomies and the accuracy of simulated corrections.
**R2C1.** The topic is highly relevant. The methodology is clearly described.

The results are presented clearly and the figures are relevant and contribute meaningfully to the manuscript.

**R2C1 Response:** Thank you for the positive assessment of our study and the manuscript.

**Reviewer 3 Comments**

Thank you for letting me review this paper. I have some general remarks followed by some point to-point questions.

**R3C1.** What was your hypothesis when setting up this study? Was it a pre planned study or were these findings during general data collection. Because with so many statistical tests you will find some significant ones.

**R3C1 Response.** We apologize for the lack of clarity. This study was exploratory in nature and was not designed to test a predefined hypothesis. In response to the reviewer’s comment regarding multiple statistical comparisons, we revised the analysis and adjusted the p-values for multiple testing using the Bonferroni correction. The purpose of the analysis was to explore potential associations rather than to establish causal relationships, and the findings should therefore be interpreted as exploratory.

Based on the received comments, we further revised the aims of this study and narrowed down the focus to the discrepancies between in vivo and simulated surgery. The aim now reads:

“*The aim of this exploratory study was to assess discrepancies between in vivo and simulated correction in 3D-planned distal radius osteotomy and to examine whether these discrepancies were associated with soft tissue- and bone density -related factors. Specifically, we explored the associations of age, bone mineral density, and planned distraction with discrepancies in volar tilt (VT), radial inclination (RI), and radial length (RL) between the simulated and in vivo surgery. We also examined the differences and agreement in residual errors between in vivo and simulated corrections.”*

We also edited the Title to reflect the changes:

“Discrepancies between in vivo and simulated correction in 3D-planned distal radius osteotomies: the influence of biological factors.”

The rest of the manuscript was revised so that the study and discussion align with the refined aims.

**R2C2**. Using correlations, the exclusion of outliers is extremely delicate, especially in such a small sample. Were the exclusions done according to a protocol? Were they done before you saw the magnitude of the errors or post hoc? Please describe how this was done.

**R3C2 Response:** Thank you for this important methodological comment. We agree that exclusion of outliers in a small sample requires particular caution. In the original version of the manuscript, outliers were identified after inspection of scatterplots and were excluded in exploratory analyses. These exclusions were not predefined in a statistical protocol and were therefore post hoc. We acknowledge that this approach may introduce bias and potentially overestimate associations. In response to this comment, we have revised the analysis to report correlation results including all observations as the primary analysis.

We have also revised the Results and Discussion sections to avoid drawing conclusions based on outlier-excluded data. Instead, potentially influential cases are discussed qualitatively in relation to clinically relevant factors that may explain measurement deviations

“*The residual VT error in IV for that participant was -8.0°, while in SIM – 3.0°. The IV error could be attributed to bone density and soft tissue tension, as the patient had low BMD (11 HU and osteopenia noted in the patient’s journal) and the planned maximum distraction was large: 13.7 mm*.”

And:

*“Again, this is due to differences in one patient who had an IV residual RL error of 2 mm, while the SIM residual error was -0.5mm. This patient had high BMD (158 HU), and the source of the residual error is not obvious.”*

**R3C3**. And why not reporting more clinical data about the patients than their HU?

**R3C3 Response:** We thank the reviewer for this suggestion. In addition to Hounsfield units, we have now included age in the analysis. During revision of the manuscript, we refined the study focus and narrowed the scope specifically to discrepancies between IV and SIM. The variables we included in the analysis are limited to those considered relevant to the IV–SIM differences.

Additional clinical variables would be more relevant in a study analysing in vivo outcomes themselves, rather than the discrepancies between in vivo (IV) and simulated (SIM) procedures. Given the refined aim, a comprehensive analysis of clinical factors influencing IV outcomes would extend beyond the scope of the present work and the available dataset.

**R3C4**. The observations are few in your spearman plots, you exclude some outliers and still the graphs do not really impress me. And all is lost if the outliers are in.

**R3C4 Response:** Thank you for this assessment. We agree that the limited sample size restricts the strength of the statistical conclusions and that correlation estimates are sensitive to influential observations in small datasets. In response to this and the R3C2 comment, we revised the analysis to include all data points in the Spearman correlation analyses. Rather than emphasizing statistical significance, we frame the findings as exploratory patterns that may inform future hypothesis-driven studies.

We also expanded the Discussion to explicitly acknowledge the limited sample size. Potential explanations for influential data points are discussed qualitatively, without excluding them from the quantitative analysis. We believe this revised presentation more accurately reflects the exploratory nature of the study and avoids overstating conclusions.

**R3C5.** Do not mix up correlations with causal relationship. If you conclude that malunions in patients with reduced bone mineral density require a larger extent of correction you anticipate a causal relationship not just an association.

**R3C5 Response:** Thank you for this important observation. We agree that our original wording risked implying a causal relationship rather than an association.

As this is an observational exploratory study based on correlation analyses, causal inferences cannot be drawn. We have carefully revised the Discussion and Conclusion sections to ensure that all findings are described as associations rather than causal effects.

The specific finding and discussion statement on a correlation between bone mineral density and the maximum planned distraction were removed because this analysis was no longer relevant to the study's refined aim.

**R3C6.** Why would volar tilt be so sensitive (and it is only 4 degrees in the graph, close to the measurement error) to bmd? I would suspect axial compression would be more sensitive but with modern locking plates any inferior bone quality is compensated given the screws are positioned correctly underneath the subchondral bone plate.

**R3C6 Response:** Thank you for this thoughtful comment. We agree that the observed difference in volar tilt is modest and approaches the range of potential measurement error. The magnitude of approximately 4° should therefore be interpreted with caution.

We do not propose a definitive biomechanical explanation for the association between residual errors and BMD. As the reviewer correctly points out, modern locking plate constructs are designed to compensate for reduced bone quality when screws are properly positioned beneath the subchondral bone. It is therefore plausible that the observed correlation reflects indirect or confounding factors rather than a direct mechanical effect of bone mineral density on correction accuracy.

Given the small sample size and exploratory nature of the study, the apparent sensitivity of volar tilt to BMD may represent a statistical artifact or the influence of individual observations. We removed this part of the analysis from the manuscript and focused instead on the discrepancies between IV and SIM.

We discussed this issue as follows:

*“The parameters investigated were associated primarily with IV-SIM discrepancies in RL rather than in VT or RI, although the largest difference in residual errors was observed for VT. One possible explanation relates to the mechanics of the osteotomy and fixation. Radial length restoration requires distraction of the distal fragment and may therefore be more sensitive to soft-tissue tension and fixation stability. In contrast, angular parameters such as VT may be more influenced by intraoperative factors such as plate positioning or fragment settling during fixation [5]. Although no clear association between the investigated parameters and VT discrepancies was observed in this study, the larger residual VT errors seen in vivo suggest that this parameter may still be susceptible to biological factors such as bone quality. Importantly, when excluding outliers, the observed VT differences remained within clinically acceptable limits in our cohort [13–16]. Nevertheless, the potential influence of low bone mineral density on VT correction accuracy may warrant further investigation in larger cohorts.”*

**R3C7.** Please report what implants were used which probably influenced the final position.

**R3C7 Response:** We thank the reviewer for this important comment. Implant characteristics are indeed relevant when analysing the accuracy of in vivo correction. We supplied the information on which plates were used in the Methods section:

*“Osteotomies were fixated using a Synthes VA-LCP two-column volar distal radius plate in 11 patients and a Stryker Variax volar distal radius plate in two patients.”*

However, in the revised manuscript we refined the aim of the study to focus specifically on the discrepancies between the in vivo (IV) and simulated (SIM) corrections. As the same plates were used in the simulations as in vivo, implant type was identical for both conditions and therefore would not influence the comparison between IV and SIM. For this reason, we did not include implant-related variables in the analysis, and state this in the Discussion:

*“Implant type was not included as a variable, as identical plates were used in the corresponding in vivo and simulated procedures.“*

**R3C8.** Did screw position relative the subchondral bone plate influence the final position? This is possible to measure

**R3C8 Response:** We agree that the distance between the screws and the subchondral bone probably influences the final position. The measurement is, however, complex, since we would have to calculate an average of the entire construct of the six to eight screws in relation to the subchondral plate in three dimensions. Such measurement would require extensive validation, which is out of the scope of this study. Moreover, our virtual planning protocol is standardised in such a way that variation in screw positioning between cases is limited.

We acknowledge this as a limitation of our study in the Discussion:

*“**A weakness of the study is that no measurements of the screw positioning relative to the subchondral plate were included and this is likely to affect the final outcome. However, since the planning was done in a standardised manner, there was little variation in screw positioning between cases. We therefore do not expect to find a significant correlation with the final result in this small, homogeneous sample.”*

**R3C9.** Volar tilt correction depends on sufficient release of the brachioradialis. Please report how the BR was managed.

**R3C9 Response:** This is an important parameter to report. The brachioradialis (BR) tendon was released at its distal insertion when required to facilitate reduction and achieve the planned sagittal correction. In cases where adequate correction could be achieved without tension from the BR, release was not performed. Based on the available operative reports, BR tendon release was documented in three patients. However, as documentation of this step was not consistently detailed across all surgical records, we cannot ensure complete capture of its use in every case. Therefore, this variable was not included in the formal analysis.

We have discussed this limitation in the Discussion:

*“Finally, the release of the brachioradialis tendon, which may influence volar tilt correction but cannot be simulated in 3D-printed models, was not included in the analysis due to variability in operative documentation.”*

**R3C10.** Is not age a useful proxy to BMD to be used to analyze your full material with more patients to see if plate type, distance of screws to subchondral bone, handling of BR, magnitude of lengthening would correlate?

**R3C10 Response:** Thank you for these suggestions. Age may indeed correlate with bone mineral density at a population level and can sometimes serve as a proxy in large epidemiological studies. However, in the present cohort, HU-based estimation of bone mineral density was available for all included patients and therefore provided a more direct, patient-specific measure than chronological age alone.

Given the limited sample size for this study (n = 13), expanding the analysis to include additional surgical variables, such as plate type or screw positioning relative to the subchondral bone, would substantially increase the risk of overfitting and spurious associations. Moreover, respective simulated and in vivo surgeries were done using the same plate system; therefore, the plate type was not a variable. While screw positioning and technical handling may influence correction accuracy, these factors were not systematically quantified and therefore cannot be reliably incorporated into our analysis.

As the revised objective of this study is to evaluate the agreement and discrepancies between simulated and in vivo correction, we chose to limit the number of predictors analyzed to a small set of variables we expect to be associated with the discrepancies. We have clarified this rationale in the Discussion and emphasized that future studies with larger cohorts could investigate additional technical parameters:

“*Future studies should employ larger cohorts of patients with varying bone mineral density values to test the effects we found in our study. Parameters, such as plate positioning and distance of screws to subchondral bone, and plate type, should also be investigated.*“

**R3C11.** Why do you use artificial bones without any BRs and DOBs and other soft tissue components or varying BMDs that you claim make a difference?

**R3C11 Response:** Thank you for this important question. The simulated procedures were intentionally performed on 3D-printed bone models without soft tissue structures to isolate the technical accuracy of the planned osteotomy and guide system under controlled conditions.

The purpose of the simulation was not to replicate the full biological environment of in vivo surgery, but rather to evaluate how accurately the surgical plan can be reproduced mechanically when factors such as soft-tissue tension, bone quality variation, and other intraoperative constraints are minimized. By removing soft tissue components (e.g., the brachioradialis tendon and distal oblique bundle), the simulation represents an “idealized” execution of the planned correction.

A simulation model incorporating these biological factors would be technically complex, and to our knowledge no validated model currently exists. In contrast, 3D-printed bone models are widely used, accessible, and suitable for comparing technical aspects of virtual surgical planning and patient-specific instrumentation.

The comparison between simulated (SIM) and in vivo (IV) outcomes is therefore central to the study objective. Any discrepancies between SIM and IV likely reflect the influence of biological and intraoperative factors absent from the simulation model, such as soft-tissue tension and bone quality. Assessing these differences can also help indicate the level of deviation to expect in real surgery when a new approach or implant design has been previously validated under simulated conditions.

We have clarified this rationale in the Introduction and further expanded it in the Discussion to acknowledge that the simulation model does not reproduce soft-tissue or biological variability:

“*Simulation models allow different surgical approaches to be tested under controlled conditions and therefore represent a promising tool for comparative studies, such as evaluating alternative PSSG designs (6,8,9). However, the simulated osteotomies were performed on 3D-printed bone models without soft-tissue structures. This simplified environment was intended to isolate the mechanical accuracy of the surgical plan and guide system.”*

**R3C12**. Page 2 Abstract

When first reading your abstract I could not figure out what the paper was about. It is not immediately obvious why bone mineral density nor maximum planned distraction would cause errors and further why you involved surgery in plastic model when you have real patients. I write this now after reading the whole article and now I understand more. So you have to write it clearer for the reader. I think you should add some form of hypothesis- why do you think these parameters would matter when setting up the study.

**R3C12 Response:** Thank you, we agree with the reviewer that the original abstract was not clear enough. In the revised abstract, we have clarified the study objective and explicitly stated the rationale for including the selected parameters. We believe the revised abstract now more clearly communicates the study’s purpose and conceptual framework:

“*Three-dimensional virtual surgical planning (3D VSP) with patient-specific surgical guides (PSSGs) can improve the accuracy of corrective distal radius osteotomy. Simulation on 3D-printed bone models allows controlled evaluation of planned corrections, but such models do not reproduce biological factors, including bone quality and soft-tissue tension. The extent to which simulated corrections reflect in vivo surgical results unclear.*

*This exploratory study included 13 patients who underwent corrective distal radius osteotomy using 3D VSP and PSSGs. Simulated osteotomies were performed on patient-specific 3D-printed bone models. Residual correction errors in volar tilt (VT), radial inclination (RI), and radial length (RL) were calculated relative to the 3D VSP for both in vivo and simulated corrections. Discrepancies between in vivo and simulated corrections were assessed, and their associations with age, bone mineral density estimated from Hounsfield units, and planned maximum distraction were explored using Spearman’s rank correlation with Bonferroni-adjusted p-values.*

*Residual correction errors in simulated osteotomies were generally comparable to those observed in vivo. The largest differences between simulated and in vivo corrections were observed for VT, although the mean difference remained within clinically acceptable limits. A strong positive correlation was found between RL discrepancy and planned maximum distraction (rₛ = 0.71, p = 0.006), and a moderate negative correlation was found between RL discrepancy and bone mineral density (rₛ = −0.64, p = 0.019). No significant associations were identified for VT or RI.*

*Simulated distal radius osteotomies on 3D-printed bone models produced correction errors broadly comparable to those achieved in vivo. However, discrepancies in RL were associated with planned maximum distraction and bone mineral density, suggesting that biological factors absent from simulation models may influence the relation between simulated and clinical outcomes. These findings support the use of simulation models for comparative studies while highlighting the need for cautious interpretation of simulation-based results.* ”

**R3C13.** Background

Page 3 line 15 One such factor is bone quality, particularly in osteoporotic patients. Bone settling in osteoporotic bone is a major concern

Why do you not measure this settling or subsidence? Report implant use- plate or something else? Bone settling a minor problem in locking plates.

**R3C13 Response:** Thank you for this comment. We agree that settling or subsidence is important, but direct measurement is not possible. Our postoperative CT scans were obtained within one week after surgery and therefore reflect immediate correction accuracy rather than secondary settling over time. As the reviewer correctly notes, the settling is directly related to the subchondral positioning of the screws and the contact area between the plate and the distal fragment in locking plates. These are all parameters that we cannot measure directly; see also our response to R3C8.

In the original Introduction section, the reference to “bone settling in osteoporotic bone” may have overstated the issue in the context of locking plate fixation. Our intention was to acknowledge that bone quality can theoretically influence surgical execution and fixation stability, rather than to imply that measurable subsidence occurred in this cohort. We have revised the Introduction section to avoid suggesting that subsidence was assessed in the present study and instead focus on the relevance of comparing in vivo and simulated results.

**R314.** Page 3 Line 19 Another parameter influencing the outcome is the planned maximum distraction to restore anatomical alignment, which may be limited by soft tissue tension.

Report how you dealt with the BR, and the dorsal soft tissue

**R3C14 Response:** Thank you for this suggestion. We agree that, since soft tissue tension is discussed as a potential limiting factor for distraction, the surgical handling of relevant soft tissues should be described explicitly. In all cases, a standard volar approach was used. The brachioradialis (BR) tendon was released at its distal insertion when intraoperative tension was judged to limit reduction or prevent achievement of the planned correction. The decision to perform BR release was made by the operating surgeon based on reduction feasibility. We justified why we did not include BR release as a variable in our study in Response to R3C9.

Regarding dorsal soft tissues, no routine dorsal release was performed. Reduction was achieved using the patient-specific guides and reduction instruments without formal dorsal capsular or intercarpal ligament release. Thus, dorsal soft tissue constraints were not surgically modified as part of the standard procedure.

**R315**. Page 4 line 1 The effect of the clinical parameters on the correction accuracy can be measured directly. You have a 4 degree VT difference according to the graph. Is that clinically important?

**R3C15 Response:** We agree that a difference of approximately 4° in volar tilt is modest and approaches the range of reported measurement variability for radiographic angular assessment. In the present study, the predefined threshold for clinical relevance was 5°, based on previous literature. The observed mean difference, therefore, falls below this threshold and should be interpreted cautiously. We did not intend to suggest that a 4° deviation is necessarily clinically meaningful in isolation.

Rather, the purpose of this analysis was to explore whether soft-tissue tension and bone-density parameters are associated with measurable discrepancies between in vivo and simulated corrections, planned virtually and performed with patient-specific surgical guides. The magnitude of these deviations and their potential clinical relevance require validation in larger cohorts with long-term outcome data.

We have revised the Discussion to clarify that the observed difference in volar tilt is small and should not be interpreted as clinically significant.

“*In contrast, angular parameters such as VT may be more influenced by intraoperative factors such as plate positioning or fragment settling during fixation. Although no clear association between the investigated parameters and VT discrepancies was observed in this study, the larger residual VT errors seen in vivo suggest that this parameter may still be susceptible to biological factors such as bone quality. Importantly, when excluding outliers, the observed VT differences remained within clinically acceptable limits in our cohort. Nevertheless, the potential influence of low bone mineral density on VT correction accuracy may warrant further investigation in larger cohorts.”*

**R3C16.** Page 5 line 11 All CT images (pre- and post-operative)…

Did you compare CT images (postOP) immediately after surgery or after healing (to measure subsidence, screw position etc)?

**R3C16 Response:** Postoperative CT scans were acquired within one week after surgery. The purpose of these scans was to assess immediate correction accuracy relative to the 3D surgical plan, not to evaluate late subsidence, implant migration, or secondary changes after healing.

Therefore, the postoperative models reflect the early mechanical result of the osteotomy and fixation rather than long-term stability or bone settling. We have clarified this explicitly in the Methods section to avoid ambiguity:

“*Postoperative CT scans were obtained within one week after surgery to evaluate immediate correction accuracy relative to the 3D virtual surgical plan; long-term subsidence or implant migration was not assessed.*“

**R3C17.** Page 5 line 21 Why specify to the lateral column? Is the maximum distance ever in the intermediate column? Or did you measure the radial/dorsal border maybe?

**R3C17 Response:** Thank you for this important anatomical clarification. In this study, planned maximum distraction was measured at the dorsal aspect of the lateral column of the distal radius, corresponding to the radial styloid region in dorsally angulated malunions. This location was chosen because, in our 3D virtual surgical planning protocol, the largest osteotomy gap in dorsally angulated deformities consistently occurred in this region.

We did not systematically measure distraction in the intermediate column, as maximum distraction was not observed there in this cohort. To improve clarity, we have revised the Methods section to specify that the measurement was performed at the dorsal–radial aspect of the osteotomy gap as defined in the 3D VSP:

“*The planned maximum distraction of the radius was defined as the largest dorsal osteotomy gap measured at the dorsal–radial aspect of the osteotomy in the lateral column (radial styloid region) [12] on the 3D VSP.*”

**R3C18.** Page 6 line 2 What does TRU stand for?

**R3C18 Response:** Thank you for pointing this out. We agree that the acronym “TRU” was unclear and potentially confusing. It originally referred to the postoperative in vivo models but was not defined explicitly in the manuscript. To improve clarity and consistency, we have removed the term “TRU” and replaced it throughout the manuscript with the clearer and explicitly defined term “IV” (in vivo). The revised text now consistently refers to postoperative models as IV models.

For example, the Methods section now reads:

“*Assessment of in vivo (IV) correction accuracy*

*IV correction accuracy was evaluated against 3D VSP (Fig. 2A) using postoperative CT-derived 3D models (IV models, Fig. 2B). Each IV model was aligned to its corresponding 3D VSP model in 3-matic (Materialise NV, Leuven, Belgium). The rigid transformation matrix describing translation and rotation between the IV model and the planned model was extracted, and residual correction errors in volar tilt (VT, °), radial inclination (RI, °), and radial length (RL, mm) were calculated relative to the 3D VSP.”*

**R3C19.** Page 6 line 17 Outliers were identified as observations that noticeably differed from general trends. Maybe there was no general trend? Maybe all the observations were simply random? I do not think you can continue to remove outliers until all of a sudden you find a trend? 1-2 removals from a data set of 13 are substantial.

**R3C19 Response:** Thank you for this important methodological comment. We agree that, in a dataset of 13 observations, excluding one or two data points may substantially influence correlation estimates and risk introducing bias if not predefined.

In the original version, outliers were identified after visual inspection of scatterplots and explored in sensitivity analyses. However, we acknowledge that this approach may create the appearance of data-driven selection.

In the revised manuscript, all primary correlation analyses are performed on the complete dataset, without excluding any observations. The previous outlier-excluded results have been removed.

**R3C20.** Page 7 Line 9 Outliers again. Did you decide to remove these because of variants in surgical techniques BEFORE knowing the values of the error? That would be the only way to do such an exclusion.

**R3C20 Response:** Thank you for pointing this out. The exclusion of outliers in the initial version of the manuscript was not predefined in a statistical protocol and was not based on surgical variants identified prior to examining the error magnitudes. Rather, these observations were identified post hoc during exploratory data inspection. We agree that, in small datasets, post hoc exclusion of data points may introduce bias and should be avoided unless based on predefined criteria. In the revised manuscript, all primary analyses are performed using the complete dataset without excluding any observations. We no longer base any conclusions on analyses with excluded data.

Potentially influential cases are now discussed qualitatively rather than being removed from the statistical analysis. The exploratory nature of the study and the limitations related to sample size have been emphasized accordingly.

**R3C21.** Page 7 Line 20 With the two outliers included, the correlation is gone as you write. So it is a delicate decision to exclude.

**R3C21 Response:** Thank you for emphasizing this important point. We agree that, in a dataset of this size, exclusion of even one or two observations can substantially alter correlation estimates and may lead to misleading conclusions.

In the revised manuscript, all primary analyses are performed using the complete dataset without exclusion of any observations. We no longer rely on correlations observed only after removing outliers, and the interpretation of the results has been moderated accordingly.

**R3C22.** Page 10 Line 8 The negative impact of low bone mineral density on the accuracy and stability of fixation of the distal radius osteotomy has been described before”. In ref 19 Ring and Jupiter write about how osteoporosis influences the radiographic outcome of a distal radius fracture, an uncontroversial topic, but do not mention the connection to radius osteotomy.

**R3C22 Response:** Thank you for this important clarification. We agree that the cited reference by Ring and Jupiter addresses the influence of osteoporosis on radiographic outcomes following distal radius fractures and does not specifically evaluate distal radius osteotomy.

Our intention was to refer more broadly to the role of bone quality in fixation stability of the distal radius. However, we acknowledge that directly extrapolating findings from fracture management to corrective osteotomy may overstate the available evidence.

In light of this and the overall revisions to the analysis, we have removed this statement from the revised manuscript and no longer address this aspect.

**R3C23.** Page 10 Line 10 …the first to explore the correlation between bone mineral density
and loss of correction in VT, RI, and RL.. I think this is because the BMD is not important anymore if you use modern locking plates.

**R3C23 Response:** We agree that modern locking plate constructs are designed to mitigate the impact of reduced bone mineral density on fixation stability. This statement, as is, is also not relevant to our refined aim.

Our intention was not to imply that bone mineral density remains a dominant determinant of correction loss with contemporary fixation systems. The purpose of including BMD was to examine whether biological factors absent in simulation models might contribute to discrepancies between simulated and actual surgical outcomes.

In response to this comment, we have removed the statement from the Discussion to avoid overstating the novelty or clinical importance. Accordingly, the Discussion has been refocused to emphasize that this study primarily evaluates discrepancies and agreement between simulated and in vivo correction, and that any observed associations with BMD should be interpreted cautiously and within the study's exploratory framework.

**R324**. Page 10 Line 14-16 We found a strong negative correlation (rs=-0.8, p=0.001) between maximum planned distraction and bone mineral density in our cohort (Fig 3), probably a consequence of more dorsal comminution and fracture collapse in patients with osteoporosis. A correlation is not a proof of a causal relationship. You cannot draw that conclusion. And there is a risk of mass significance with so many statistical tests.

**R3C24 Response:** Thank you for this important comment. We agree that correlation does not imply causation, and our original wording may have overstated the interpretation of the observed association. We have revised the Discussion to remove causal language, and we explicitly acknowledge that the study design does not allow causal inference:

“*Given the exploratory nature of the study and the number of correlations examined, these findings should be interpreted cautiously and considered hypothesis-generating.”*

Regarding the concern about multiple statistical testing, we agree that the number of correlations explored increases the risk of type I error. We adjusted p-values for multiple testing and highlighted the exploratory, hypothesis-generating design of our study.

**R2C25.** Page 10 Line22 …leading to settling of the distal fragment on the distal screws of the plate, which primarily leads to rotation and loss in VT. If you measure the distance of the distal screws to the subchondral bone you will know if this hypothesis is plausible. You can plot the distance vs the HU but you can also compare the distance immediately postop vs after osteotomy healing.

**R3C25 Response:** Thank you for this technically relevant comment. We agree that evaluating the distance between the distal screws and the subchondral bone and comparing immediate postoperative alignment with alignment after healing would be necessary to directly test the hypothesis of distal fragment settling on the plate. However, this was not the aim of the study (which we revised to improve clarity). We also explained that the present study relied on the in vivo correction accuracy relative to the virtual plan taken within one week after surgery, to enable comparison with the simulated surgery, in which the healing cannot be simulated.

We acknowledge that our original wording may have implied a mechanistic explanation that was not directly investigated. We have revised the Discussion to avoid speculative language regarding potential mechanisms that could explain the discrepancies between in vivo and simulated surgery.

R326. Page 11 line 2 Furthermore, using plates with variable screw angles could contribute to the errors as opposed to fixed-angle plates.

Interesting -Is it so? Do you have a reference for that? It is a source of technical error but in this study the surgeons are experienced. But agree- i prefer to use fixed angle plates for this.

**R3C26 Response:** We agree that our original statement regarding variable-angle plates may have been speculative and insufficiently supported by direct evidence. In this cohort, all patients were treated using volar locking plate system with variable-angle screw capability. We did not compare implant types, nor did we systematically evaluate the influence of screw angulation freedom on correction accuracy. Therefore, we cannot conclude that the use of variable-angle plates contributed to the observed deviations.

While variable-angle constructs theoretically allow greater intraoperative flexibility, they may also introduce technical variability. However, given the experience of the operating surgeons and the absence of a comparative implant analysis, we agree that attributing errors to plate design is not justified.

We have therefore revised the Discussion to remove this speculative statement and now refer more generally to potential technical factors without implicating a specific implant design.

**R327.** Page 11 Line 6 To minimize the residual errors in osteoporotic patients, 3D-printed, patient-specific plates could be considered

That is a wild guess. Why would it reduce the errors? And do refs 20 and 21 really discuss the residual errors?

**R3C27 Response:** Thank you for highlighting this point. We agree that the statement regarding patient-specific plates was speculative and not directly supported by the findings of the present study. Our intention was to suggest a potential direction for future research rather than to imply evidence of reduced residual correction errors.

However, as our study did not evaluate implant design or compare fixation strategies, this statement was not sufficiently justified and has been removed from the revised manuscript. The associated references have also been removed.

**R3C28.** Page 11 line 18 The secondary aim of this study was to compare the residual errors between actual and simulated surgeries.

This part is difficult to interpret without knowing if you compared the model to the surgical result immediately postop or after healing.

**R3C28 Response:** Thank you for this comment. We agree that this was not clear in the original manuscript. As noted in our response to Comment R3C16, the residual errors for the in vivo procedures were calculated using the immediate postoperative CT scans, prior to any healing or remodeling. Therefore, the comparison between simulated and in vivo corrections reflects the accuracy of the surgical execution rather than postoperative biological changes.

**R3C29**. Page 12 line 6 The major limitation of this study is the small cohort… Here you list all the reasons I have raised that the results of your study maybe not are robust enough. I think they are interesting but then need to be tested in a prospective protocol and with a more homogenous material. I do not think, however, you will find any correlation between BMD and VT in the plate fixed if the screws are placed correctly.

**R3C29 Response:** Thank you for this constructive perspective. We agree that the small sample size and heterogeneity limit the robustness of the findings and that prospective validation in a larger, more homogeneous cohort would be necessary to test any hypothesis derived from this exploratory analysis.

Based on your and other reviewers’ comments, we acknowledge that the original focus of the study was too broad. Our intention was not to establish a definitive relationship between bone mineral density and volar tilt correction, but rather to explore whether measurable associations exist that explain differences between simulated and in vivo corrections. Still, given the small cohort, the results should be interpreted cautiously. We have revised our analyses and the Discussion to narrow the focus to the discrepancies between simulated and in vivo surgeries and to present the results as hypothesis-generating observations that may guide the design of prospective investigations.
