# Supplementary material for: Exploring discrepancies between in vivo and simulated correction in 3D-planned distal radius osteotomies: the influence of biological factors
Source: PLoS One. 2026 May 26;21(5):e0350018. doi: 10.1371/journal.pone.0350018 (PMC13210242; doi:10.1371/journal.pone.0350018)
Supplement: Attachment — Submitted filename: PONE rev 1 reviewer reply.docx [file pone.0350018.s003.docx]

Thank you for extensive work with the paper. Most of the text has been replaced and I had to ask for a clean sheet without changes to be able to read. But the paper has improved, is much easier tyo read and more focused on what you have found and is able to conclude upon.

If you see it as a limitation with the low number of patients, still do so many statistical test that you have to use bonferoni, exclude ”outliers” and cannot really explain the error in VT, I would suggest you just report this as an exploratory study, show differences as 95% CI between simulation and saw bones and refrain from any statistical analyses? The study is not powered to show any differences.

I still have some questions as listed below below, but far less than the first time. The page and line numbers correspond to the the new clean paper without changes.

Line 8 You list technical factors such *as guide-to-bone fit [6], the design of the PSSG and the resolution of the CT images used for planning* as obstacles for clinical studies due to variation between patients. But why would 3D printed patient specific models make that more simple/reliable. The errors would be the same? And as you write you introduce new sources of errors like soft tissue tension bmc/d etc.

Page 5 Line 12 Was HU extracted from the preop CT images, that is no metal artefacts.

Page 6 line 5 Were the plate brand chosen because they fitted best in the planning or was that just surgeons choice?

Page 7 line16  *this method is robust to small sample sizes and does not assume normality*

But then you cannot exclude outliers that does not ”fit” to the expected line.

Page 8 line 3; 83.8 HU (range 5.8–189.6). Do you need decimals here?

Page 8 line ; 4.5 to 13.7mm Same here;. was your measurment accuracy at that level? Please check the rest of the results and discussion sections if all decimals are necessary (on line 18 you even report the measurement error on a micrometer scale (0.41° and -0.29mm).

Page 11 line 1 *Radial length restoration requires distraction of the distal fragment and may therefore be more sensitive to soft-tissue tension*.

Did you detach the BR? Not written in methods?

And still distance of distal screws to subchondral bone is measurable and may explain the influence of the bone density? It is mention a little later as a limitation but why do you not simply measure it ? It is only 13 patients? And plate position- look thru the xrays- maybe that can explain the outliers?

Page 11 line 3 And why would low bone density correlate to angular error more than length errors. I think the latter. That makes sense just as RL and planned distraction.

Page 11 line 11 line 7 *when excluding outliers,*

Still not sure you can do like this- exclude ”outliers” that do not fit
