# Supplementary material for: Exploring discrepancies between in vivo and simulated correction in 3D-planned distal radius osteotomies: the influence of biological factors
Source: PLoS One. 2026 May 26;21(5):e0350018. doi: 10.1371/journal.pone.0350018 (PMC13210242; doi:10.1371/journal.pone.0350018)
Supplement: Attachment — Submitted filename: Response to Reviewers_round 2.docx [file pone.0350018.s005.docx]

Dear Editor and Reviewers,

On behalf of all authors, we thank you for your further suggestions, which we have addressed, as shown below. In this response letter, we cite the comments in full and refer to the comments using the following notation: R3C1 denotes Reviewer 3, Comment 1, and so on. All corresponding revisions in the manuscript have been clearly indicated using this convention.

**R3C1:** Thank you for extensive work with the paper. Most of the text has been replaced and I had to ask for a clean sheet without changes to be able to read. But the paper has improved, is much easier tyo read and more focused on what you have found and is able to conclude upon.

**R3C1 Response:** Thank you for your positive assessment and such a thorough and nuanced review of our manuscript. We believe that addressing your comments further improved the manuscript.

**R3C2:** If you see it as a limitation with the low number of patients, still do so many statistical test that you have to use bonferoni, exclude ”outliers” and cannot really explain the error in VT, I would suggest you just report this as an exploratory study, show differences as 95% CI between simulation and saw bones and refrain from any statistical analyses? The study is not powered to show any differences.

**R3C2 Response:** We thank the reviewer for this helpful comment. We have strengthened the exploratory framing throughout the manuscript, including adding "Exploring discrepancies…" to the title, and have removed the Bonferroni correction and report 95% confidence intervals for each r_s_ value alongside uncorrected p-values. All associations are explicitly framed as hypothesis-generating and are tested on all available data. We edited the Statistical analysis section (p.7) as follows:

*“Spearman’s rank correlation coefficient (r_s_) and corresponding 95% confidence intervals were calculated for each pair of variables, as this method is robust to small sample sizes and does not assume normality. Given the exploratory nature of the study, p-values were reported alongside effect sizes for completeness. All associations should be interpreted as hypothesis-generating.”*

Accordingly, we revised the results section and Fig. 3 and avoided stating that any correlation was significant (or strong or moderate, due to wide CI intervals).

**R3C3:** I still have some questions as listed below below, but far less than the first time. The page and line numbers correspond to the the new clean paper without changes.

Line 8 You list technical factors such *as guide-to-bone fit [6], the design of the PSSG and the resolution of the CT images used for planning* as obstacles for clinical studies due to variation between patients. But why would 3D printed patient specific models make that more simple/reliable. The errors would be the same? And as you write you introduce new sources of errors like soft tissue tension bmc/d etc.

**R3C3 Response:** We thank the reviewer for this observation and agree that the text was unclear. The advantage of 3D-printed models is not that they eliminate technical sources of error, but that they allow the same deformity to be used repeatedly under controlled conditions. For example, to compare two different guide designs without the confound of patient-specific anatomical variation. We agree that technical errors such as guide-to-bone fit and CT resolution apply equally to simulation, and we have revised the text to make this distinction explicit. We also acknowledge, as the reviewer notes, that simulation introduces its own limitations by removing biological factors such as soft-tissue tension and bone quality, which is precisely the motivation for the present study. We revised the Introduction paragraph as follows:

*“Surgical accuracy may be influenced by technical factors such as guide-to-bone fit [6], the design of the PSSG [3,5], and the resolution of the CT images used for planning [7]. Evaluating the relative contribution of such factors is difficult in clinical studies because each patient presents with a unique combination of anatomy, bone quality, and deformity. This inter-patient variability makes it challenging to isolate the effect of a single technical factor, for example, two guide designs, without confounding from patient-specific differences. Patient-specific 3D-printed bone models, which have been shown to reproduce bony surface anatomy accurately [10], provide a potential solution.”*

**R3C4:** Page 5 Line 12 Was HU extracted from the preop CT images, that is no metal artefacts.

**R3C4 Response:** That’s correct, the HU value was extracted from preoperative CT, without metal artifacts. We clarified this in the text:

*”The distal ulna was selected because it was consistently included in all preoperative CT scans and is anatomically adjacent to the surgical site. No metal artifacts were present in the region of interest.”*

**R3C5:** Page 6 line 5 Were the plate brand chosen because they fitted best in the planning or was that just surgeons choice?

**R3C5 Response:** Thank you for this question. The choice of plates was dictated by the surgeon’s preference, and the availability of STL files provided by the manufacturer. The information was added in the manuscript, under Assessment of in vivo (IV) correction accuracy section:

*“The type of plate was dictated by the surgeon’s preference, the availability of STL files, and the plate fit on the bone during virtual planning. The decision to release the brachioradialis was made at the operating surgeon's discretion but was not documented consistently.”*

**R3C6:** Page 7 line16  *this method is robust to small sample sizes and does not assume normality*

But then you cannot exclude outliers that does not ”fit” to the expected line.

**R3C6 Response:** We thank the reviewer for this comment. We agree and confirm that all 13 patients are included in every analysis, and no data points are excluded. Individual patients are described narratively in the context of the Bland-Altman analysis to aid clinical interpretation of the limits of agreement, but this does not affect the statistical results.

**R3C7:** Page 8 line 3; 83.8 HU (range 5.8–189.6). Do you need decimals here?

**R3C8:** Page 8 line ; 4.5 to 13.7mm Same here;. was your measurment accuracy at that level? Please check the rest of the results and discussion sections if all decimals are necessary (on line 18 you even report the measurement error on a micrometer scale (0.41° and -0.29mm).

**R3C7 and 8 Response:** Thank you for pointing this out. We reviewed the manuscript to ensure that all decimals are consistently used and necessary as follows:
Means and ranges: one decimal

Correlation coefficients: two decimals

Bland-Altman bias and limits of agreement: one decimal

Age: whole numbers

HU values: whole numbers.

**R3C9:** Page 11 line 1 *Radial length restoration requires distraction of the distal fragment and may therefore be more sensitive to soft-tissue tension*. I think the detachment or not of the BR is super important to allow correction. Write out that it was not documented. It is ok. But the reader wants to know.

**R3C9 Response:** We agree and thank the reviewer for raising this point. Brachioradialis (BR) release reduces deforming soft-tissue tension and may facilitate radial length restoration and volar tilt correction. Whether BR release was performed was at the operating surgeon's discretion and was not consistently documented, and could therefore not be included in the analysis. We have added the following statement in the Assessment of in vivo correction accuracy section:

*”The decision to release the brachioradialis was made at the operating surgeon's discretion but was not documented consistently. ”*

**R3C10:** And still distance of distal screws to subchondral bone is measurable and may explain the influence of the bone density? It is mention a little later as a limitation but why do you not simply measure it ? It is only 13 patients? You have CT data and access to mathematically skilled people. And plate position- look thru the xrays- maybe that can explain the outliers?

**R3C10 Response:** We agree that screw distance to the subchondral bone may influence the in vivo correction outcome. However, one would first need to define and validate a summary measure across a construct comprising several screws in three dimensions to obtain reliable data. Furthermore, the outcome of interest in this study is the discrepancy between in vivo and simulated corrections, not the absolute in vivo accuracy. Since the 3D-printed models do not replicate the properties of bone, including the subchondral plate, screw positioning relative to the subchondral bone cannot be meaningfully assessed or compared in the simulated setting. This variable, therefore, cannot explain IV–SIM discrepancies and falls outside the scope of the present analysis. We agree it is relevant to studies of absolute in vivo correction accuracy and have noted this in the limitations:

*”A weakness of the study is that no measurements of the screw positioning relative to the subchondral plate were included, which may influence the absolute in vivo correction outcome. However, meaningfully assessing screw positioning relative to the subchondral bone in a simulated setting is not feasible because the 3D-printed models cannot replicate bone properties.”*

**R3C11:** Page 11 line 3 And why would low bone density correlate to angular error more than length errors. I think the latter. That makes sense just as RL and planned distraction.

**R3C10 Response:** We thank the reviewer for this comment. On reflection, we agree that our earlier speculation linking VT errors to bone quality was not well supported by our results and have removed it. Both RL and VT discrepancies are likely influenced by soft-tissue tension acting on the distal fragment during fixation, a factor that cannot be reproduced in the simulated setting. While correlations were observed between RL discrepancies and planned maximum distraction and BMD, we note that RL and VT are not independent. The loss of VT correction will also shorten RL. Given this interdependence and the wide confidence intervals, we have been careful not to overinterpret these associations:

*“The observed IV-SIM discrepancies in both RL and VT may reflect intraoperative factors not captured in the simulation. Both RL and VT restoration require distraction of the distal fragment and may therefore be sensitive to soft-tissue tension and fixation stability. The systematic difference observed in VT suggests that intraoperative factors such as bone settling due to plate offset from the bone [5] are at play in vivo but not in SIM. Importantly, however, the interquartile range of the observed VT differences remained within clinically acceptable limits in our cohort [13–16]. Correlations were observed between RL discrepancies and both planned maximum distraction and BMD, though these should be interpreted with caution, given the wide confidence intervals and the interdependence between RL and VT, where loss of VT correction will also affect RL.”*

**R3C11:** Page 11 line 11 line 7 *when excluding outliers,*

Still not sure you can do like this- exclude ”outliers” that do not fit

**R3C11 Response.** We thank the reviewer for this observation. To clarify, no outliers are excluded from any analysis. The original phrasing was misleading, as it referred to the visual representation in the box-and-whisker plot, where individual points outside the interquartile range are displayed as separate markers by convention, not excluded from the statistics. We have removed the ambiguous phrasing and replaced it with:

*”Importantly, the interquartile range of the observed VT differences remained within clinically acceptable limits in our cohort [13–16].”*
